# Development of a Rapid Method for Residence Time Distribution Measurement in Twin-Screw Wet Granulation Based on Image Processing with Lab Color Space

**DOI:** 10.3390/pharmaceutics17070929

**Published:** 2025-07-18

**Authors:** Jie Zhao, Geng Tian, Ying Tian, Haibin Qu

**Affiliations:** Pharmaceutical Informatics Institute, College of Pharmaceutical Sciences, Zhejiang University, 866 Yuhangtang Road, Hangzhou 310058, China; zhaojie_1021@zju.edu.cn (J.Z.); iamtiangeng@zju.edu.cn (G.T.); 12019040@zju.edu.cn (Y.T.)

**Keywords:** continuous manufacturing (CM), twin-screw wet granulation (TSWG), image processing, mean residence time (MRT), tracer method

## Abstract

**Background/Objectives**: In the twin-screw wet granulation (TSWG) process, accurate measurement of residence time distribution (RTD) is critical, as it characterizes material transport kinetics and mixing behavior. It plays a critical role in evaluating the homogeneity and stability of the granulation process and optimizing process parameters. It is necessary to overcome the limitations arising from the complex and time-consuming procedures of conventional RTD determination methods. **Methods:** This study proposes a new RTD detection method based on image processing. It uses black dye as a tracer to obtain RTD curve data, and the effects of process parameters such as tracer dosage, screw speed, and feeding rate on the RTD were investigated. **Results:** The results show that the established method can accurately determine RTD and that the tracer dosage has no significant effect on the detection results. Further analysis revealed that the screw speed is negatively correlated with the mean residence time (MRT). As the speed increases, not only does the MRT shorten, but its distribution also decreases. Similarly, an increase in the feeding rate also leads to a decrease in the MRT and distribution, but it is worth noting that lower feeding rates are beneficial for achieving a state close to mixed flow, while excessively high feeding rates are not conducive to sufficient mixing of materials in the extruder. **Conclusions:** The RTD detection method provides a reliable parameter basis and theoretical guidance for the in-depth study of the TSWG process and the development of quality control strategies.

## 1. Introduction

Continuous twin-screw wet granulation (TSWG) technology, as an innovative alternative to traditional batch granulation, has become a potential approach to improve granulation efficiency and product quality in the pharmaceutical and chemical industries, owing to its inherent advantages of high efficiency and precise controllability [1,2]. A twin-screw continuous granulator is typically composed of two co-rotating, self-wiping screws enclosed in a barrel, along with kneading and conveying elements [3]. In the TSWG process, the raw materials are fed into the granulator from the powder feeding port and are mixed and transported along the screw direction under the action of the conveying elements. They come into contact with the liquid injected from the liquid feeding port and are wetted into blocks, forming low-strength, wet aggregates inside the granulator. Subsequently, the combined shearing and kneading actions of the screws break down the initial aggregates, promoting uniform granule growth through layering of primary powder onto the wetted nuclei [4,5].

The TSWG process exhibits complex phenomena, including material backmixing behavior, laminar flow dominance within the barrel, and the presence of a dead zone or short-circuiting effects due to equipment geometry. These factors contribute to a distribution of residence times for the material within the barrel, which forms the residence time distribution (RTD) [6,7]. The RTD, as a key parameter for quantifying material flow in continuous systems, not only characterizes the mixing efficiency and homogeneity of the process but also provides crucial information for process control of continuous production. Specifically, RTD data can be employed to analyze the propagation mechanisms of process disturbances, identify deviations from the desired control state, determine material traceability for root-cause analysis, and establish diversion strategies to isolate non-compliant materials. This provides a reliable decision-making basis for quality control in continuous manufacturing (CM) processes [8].

The RTD is typically characterized by the density function (E(t)) and the cumulative residence time distribution function (F(t)). E(t) represents the probability density of substances leaving the system at a certain moment t. F(t) represents the fraction of material that has resided in the system for a time less than or equal to t and is defined as the integral of E(t) from 0 to t [9]. The calculation formulas for E(t) and F(t) are shown in Equations (1) and (2), where C_i_ is the tracer concentration at the outlet at time t_i_, which represents the *i*-th sampling time used for the tracer. Research has shown that RTD characteristics are influenced by multiple factors, such as material properties, process parameters (screw speed and feeding rate), and equipment geometry (screw element combination and L/D (length to diameter) ratio of the element) [1,10,11]. Traditional RTD measurements, which employ pulse or step input of tracers and subsequent online monitoring or offline analysis, present limitations in terms of real-time performance, applicability, and operational efficiency.

(1) $$E(t)=\frac{C(t)}{\int_{0}^{\infty }C(t)\mathrm{dt}}=\frac{C_{i}}{\sum_{0}^{n}C_{i}\Delta t_{i}}$$

(2) $$F(t)=\int_{0}^{t}E(t)\mathrm{dt}=\frac{\int_{0}^{t}C(t)\mathrm{dt}}{\int_{0}^{\infty }C(t)\mathrm{dt}\sum_{0}^{n}C_{i}\Delta t_{i}}=\frac{\sum_{0}^{t}C_{i}\Delta t_{i}}{\sum_{0}^{n}C_{i}\Delta t_{i}}$$

Primary RTD detection methods for the TSWG process include high-performance liquid chromatography (HPLC) [12], imaging techniques [13,14], near-infrared (NIR) spectroscopy [15], chemical imaging (NIR-CI) [5], fluorescence methods [16], and positron emission particle tracking [17]. Among these, the HPLC method requires complex preprocessing steps for the sample, making it difficult to achieve online detection and unable to identify RTD changes caused by materials and processes variations in a timely manner. Additionally, fluorescence and positron emission particle tracking methods require expensive optical detection equipment, and the use of tracers may affect data reliability due to potential photodegradation.

Recent advances in image processing technology offer promising methods for characterizing RTD in twin-screw granulation, enabling non-invasive and high-resolution analysis of powder mixing behavior and uniformity. Haser et al. [13] employed an image-based method to measure the RTD of materials within a twin-screw granulator. Following a pulse injection of tracer at the extruder inlet, a high-speed camera continuously captured particle-flow video at the outlet. By analyzing the captured images, an RGB intensity curve was extracted. The RTD density function (E(t)) was then fitted to this curve, with RGB intensity serving as a proxy for tracer concentration based on a calibration curve relating RGB intensity to known tracer concentrations.

However, determining the concentration based on the RGB color of materials at the outlet in RTD analysis has limitations and introduces risks of calculation errors. Specifically, fluctuations in material throughput can lead to variations in the observed RGB intensity, independent of the tracer concentration. Similarly, the degree of tracer dispersion can affect the measured RGB intensity, potentially leading to inaccurate concentration estimations. Furthermore, the rotating screws can introduce artifacts in the captured images, such as motion blur or reflections, which further complicate the color analysis and introduce errors into concentration calculations.

The Lab color space is a device-independent standardized color model that separates brightness (L*) from chromaticity (a* and b* channels). In this model, the L* channel represents brightness, while the a* and b* channels represent color information. Each combination of values corresponds to a specific color that is independent of the device. This standardized color space enables effective comparison, simulation, and matching of colors across different devices, which is essential for applications in digital imaging [18]. A non-destructive and efficient image processing method was developed to measure residence time distribution (RTD) in a laboratory extrusion process, as well as the effects of screw speed and barrel temperature on RTD, as determined by the image processing method [19]. Color analysis can also be used to detect concentration content. A machine vision-based method was developed to indirectly and accurately quantify ultralow drug content in real time during TSWG by using the b* value from color analysis to determine granule drug content [20,21].

This study proposes an image processing method for RTD analysis in the TSWG process, addressing critical limitations including outlet-image acquisition interference and inaccurate concentration measurement based on the RGB color space. The proposed methodology systematically integrates pulse response techniques with image analysis based on the Lab color space. It utilizes glass tubes for particle collection from the outlet during the TSWG process and performs concentration analysis of the static imaging of the particles. Specifically, solvent black 5 is used as a tracer for its distinct color contrast against material, enabling reliable material differentiation. By capturing and analyzing the particle images collected from the TSWG process, an image-based RTD measurement method is established. Furthermore, the effects of different tracer dosages, screw speeds, and feeding rates on the RTD of the material are analyzed.

## 2. Materials and Methods

### 2.1. Materials

The experimental setup utilized a pharma 11 twin-screw extruder (Thermo Fisher Scientific, Waltham, MA, USA) equipped with a CA-1116A EYELA cooling water circulation device (Tokyo Physical and Chemical Equipment Co., Ltd., Tokyo, Japan). A V-2 mixer (Wuxi Chenli Powder Equipment Co., Ltd., Wuxi, China) and a single-screw volumetric feeder (Shanghai Qinrui E-commerce Co., Ltd., Shanghai, China) were used for material preparation. A BL 100 flow peristaltic pump equipped with a YZ15 pump head (Changzhou Weixier Fluid Technology Co., Ltd., Changzhou, China) and a silicone hose (2.4 mm × 5.6 mm, 19 #) was employed. An XS 105 Analytical Balance (Mettler Toledo Instruments, Columbus, OH, USA) and a TC3K electronic balance (Changshu Shuangjie Testing Instrument Factory, Changshu, China) were used for precise weight measurements. Images were captured using a P30 Pro camera (Huawei Technologies Co., Ltd., Shenzhen, China). All experiments were conducted using the Milli-Q Ultra Pure Water System (Millipore, Burlington, MA, USA).

Anhydrous lactose (lot No. 1320020470) and microcrystalline cellulose (lot No. 20200719), both provided by Infinitus (China) Co., Ltd. (Guangzhou, China), and Polyvinylpyrrolidone K30 (PVP K30, ISP Technologies, Inc. (Waterford, MI, USA), Lot 0001893639) were used. Solvent black 5 (Shanghai Xianding Biotechnology Co., Ltd. (Shanghai, China), Lot KPXRXDT) was utilized as a tracer component. Deionized water was prepared by an ultrapure water system.

### 2.2. Granulation Experiments

The formulation used in this study consists of 85% (*w*/*w*) anhydrous lactose, 10% (*w*/*w*) microcrystalline cellulose, and 5% (*w*/*w*) PVP K30. The raw materials were weighed according to the prescribed ratios and blended in a V-shaped mixer for 60 min to achieve a homogeneous premix before the subsequent processing steps. This premix was then fed into a Pharma 11 twin-screw extruder via a single-screw volumetric feeder.

**Figure 1:**
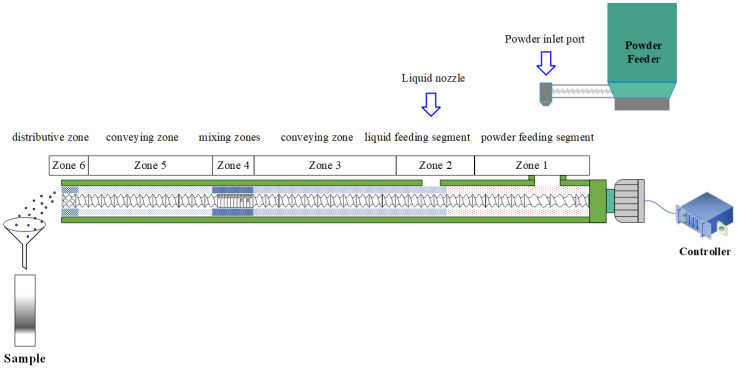
Setup for the experiments.

The setup for the experiments is illustrated in Figure 1. The screw configuration comprises two conveying zones, a kneading zone, and a discharge zone. The conveying zone consists of two types of feed screw elements, i.e., a long-helix feed screw (2 L/D) and a feed screw (1 L/D). The kneading zone is composed of a series of mixing elements with 90° and 0° staggering angles, followed by forward-staggered elements at 30° and 60°. This configuration promotes distributive mixing while minimizing shear force. A distributive feed-screw element at the discharge end of the granulator barrel breaks oversized agglomerates. The quantities and arrangement of each element are detailed in Figure 1.

The premix was fed into the inlet of the first zone of the extruder using a volumetric feeder. Deionized water was used as the granulation binder and was injected into the extruder through a liquid injection converter using a peristaltic pump. The barrel temperature was maintained at 25 °C throughout the TSWG process. Different process parameters, including tracer dosage, screw speed, and feed rate, were adjusted according to the specific experimental objectives of the TSWG process.

**Figure 2:**
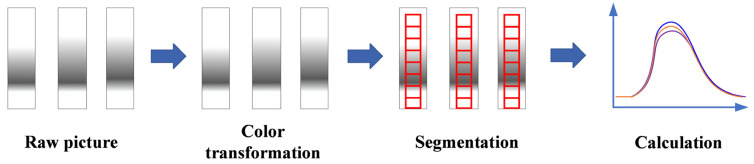
Schematic diagram of image processing for RTD calculation.

Solvent black 5 was used as the tracer to determine the RTD of the TSWG process. After the granulation process reached a steady state, as indicated by a constant torque value, the prepared tracer pulse was introduced into the extruder through the powder feed port. The time of tracer injection was recorded as the start time (T_0_). Granules exiting the discharge port were collected using a glass tube with a diameter of 40 mm and a height of 265 mm. The collection process continued until the granules were free of tracer and the particles approached the top of the tube, as confirmed by reversion of the material color to its original white. The end time of the collection period was recorded as T_1_. Images of the glass tube with graduated sample were captured using the rear camera of a P30 Pro with a resolution of 2340 × 1080. The distance between the camera and the object being measured was 1 m, and the camera uses a 1× zoom. The images were then processed using image processing algorithms to determine the concentration of the tracer. The image processing step is shown in Figure 2. These data were used to construct the RTD curve and calculate the relevant parameters of the RTD.

### 2.3. RTD Model Development Based on Image Processing

This study employed the pulse response method to measure the RTD and its parameters in the TSWG process. RTD calculation involved four main steps, including the definition of the detection region, performing grid division, calculating the lightness value (L*), and determining the RTD functions (E(t) and F(t)). These steps were designed to ensure accurate and reliable RTD parameter estimation.

#### 2.3.1. Definition of the Detection Region

For the obtained sample images, the detection region was defined from the bottom of the glass tube to the top of the granules. The width of this detection region was consistently fixed at 100 pixels to ensure uniformity across measurements. To minimize interference from light-source reflections, the central part of the measuring cylinder, where no significant reflections were observed, was chosen as the detection region. To further reduce the influence of material color on the calculation results, a blank sample group without any added black tracer was used as the background. This blank sample served as a baseline for calculation of the RTD curve by compensating for any color variations inherent to the materials. The detection region and background configuration ensured that the calculated RTD functions (E(t) and F(t)) accurately reflected the dynamics of the TSWG process without external distortions.

#### 2.3.2. Grid Division and Calculating L*

The selected detection region was uniformly divided into 200 grids along its height. For each rectangular grid area, the characteristic values reflecting the tracer concentration were calculated. These values were derived from the lightness (L*) in the Lab color space, which was obtained after processing the granule sample images. The captured images were initially in the RGB color format. To reduce the influence of color on RTD measurement, the RGB color mode of the images was converted to the Lab color space. In the Lab color space, L* represents the lightness of a pixel, with a value range of [0, 100], indicating pure black to pure white. The *a* value represents the range from red to green, with a value range of [−128, 127], and *b* represents the range from yellow to blue, with a value range of [−128, 127]. However, a direct conversion from the RGB color space to the Lab color space is not possible. Instead, an intermediate conversion to the XYZ color space is required. The conversion process consists of two steps. First, the RGB color space is converted to the XYZ color space; then, the XYZ color space is converted to the Lab color space. The relationship between the RGB and XYZ color spaces is defined by the following equation [22].

(3) $$\left[\begin{array}{c} X\\ Y\\ Z \end{array}\right]=\left[\begin{array}{ccc} 0.412453 & 0.357580 & 0.180423\\ 0.212671 & 0.715160 & 0.072169\\ 0.019334 & 0.119193 & 0.950227 \end{array}\right]\;\times \;\left[\begin{array}{c} R\\ G\\ B \end{array}\right]$$

where *R*, *G*, and *B* are the values of the three channels in the RGB color space and *X*, *Y*, and *Z* are the values in the XYZ color space.

Once the RGB color space is converted to the XYZ color space, the values are further transformed into the Lab color space. The conversion formula from the XYZ color space to the Lab color space is expressed using the following equations [23].

(4) $$f(t)=\left\{\begin{array}{cc} t^{\frac{1}{3}} & \mathrm{if}\;t>{\left(\frac{6}{29}\right)}^{3}\\ \frac{1}{3}{\left(\frac{29}{6}\right)}^{2}t+\frac{4}{29} & \mathrm{otherwise} \end{array}\right.$$

(5) $$L^{*}=116f(Y)-16\;a^{*}=500\left[f(X)-f(Y)\right]\;b^{*}=200\left[f(Y)-f(Z)\right]$$

In the formula, L*, a*, and b* are the three channels of the final Lab color space, and they serve as the basis for further calculations. The lightness value (L*) was specifically used to reflect the tracer concentration in the RTD calculation.

#### 2.3.3. Determining the RTD Functions (E(t) and F(t))

**Figure 3:**
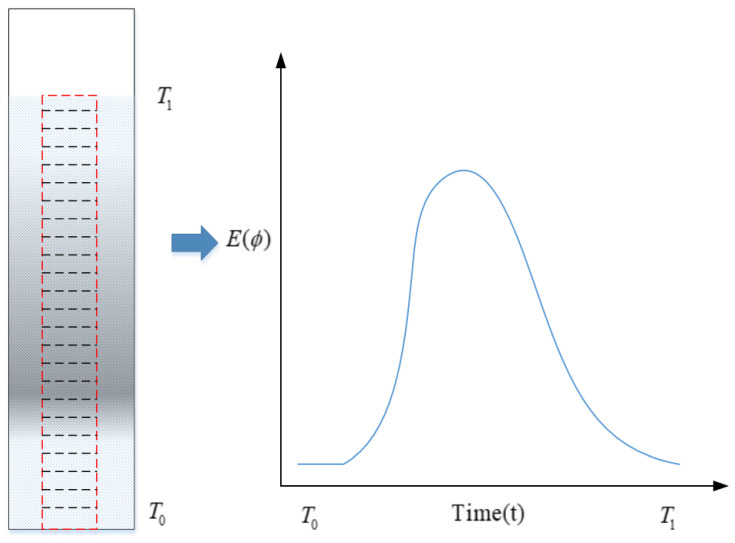
Schematic diagram of image-based RTD measurement.

The brightness level of a tracer shows a negative correlation with its concentration. By dividing the tracer concentration for each sample by the total tracer concentration, the brightness value curve is normalized. This normalization provides the percentage of tracer at different times, corresponding to the RTD functions (E(t) and F(t)). The measurement principle is illustrated in Figure 3.

The mean residence time (MRT, *t_m_*) quantitatively reflects the residence time of materials in the barrel. The MRT (*t_m_*) describes the time required for 50% of particles to exit the system after their entry into the system at T_0_. The variance ($\sigma^{2}$) reflects the degree of dispersion in the RTD. The calculation formulas for *t_m_* and $\sigma^{2}$ are provided in Equations (6) and (7) [23].

(6) $$t_{m}=\frac{\int_{0}^{\infty }tE(t)dt}{\int_{0}^{\infty }E(t)dt}=\int_{0}^{\infty }tE(t)dt$$

(7) $$\sigma^{2}=\int_{0}^{\infty }{\left(t-t_{m}\right)}^{2}E(t)dt$$

The MRT can be used to assess the degree of mixing, where an increased MRT indicates more thorough mixing of materials in the barrel. In the production process, the real-time data from online process analysis instruments are used to analyze the operating status of the continuous granulation process. When deviations in material properties are detected, the corresponding materials are emptied based on the MRT value.

To address discrepancies caused by different time units and to facilitate comparison under different conditions, a dimensionless residence time (θ) is often defined to replace time *t*. The dimensionless variance (σ_θ_^2^), which compares the time distribution, is calculated as shown in Equation (8).

(8) $${\sigma_{\theta }}^{2}=\frac{\sigma^{2}}{{t_{m}}^{2}}$$

The flow state in the reactor can be determined based on the shape of the RTD curve. For an ideal piston-flow reactor, the flow state corresponds to σ_θ_^2^ = 0, indicating perfect uniformity. For a fully mixed ideal flow reactor, the flow state corresponds to σ_θ_^2^ = 1, indicating maximum mixing. Intermediate values (0 < σ_θ_^2^ < 1) indicate a flow state in the reactor that is between piston flow and mixed flow.

## 3. Results and Discussion

### 3.1. Calibration and Error Analysis of Powder Feeding Rate and Liquid Feeding Rate

#### 3.1.1. Calibration and Error Analysis of Powder Feeding Rate

**Figure 4:**
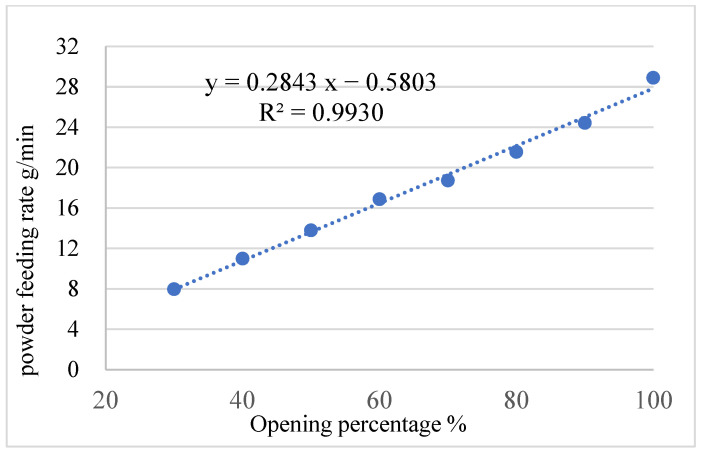
Relationship between different opening percentages and powder feeding rates of volumetric feeders.

To ensure consistent granulation conditions, it is crucial to calibrate the feeding rate and evaluate its errors. This section focuses on the calibration and error analysis of the powder feeding rate in a TSWG system, as the accuracy and stability of the feeding rate have a significant impact on the continuous granulation process. The feeding flow rate was controlled by adjusting the opening percentage of the volumetric feeder. The mass of the raw materials remaining in the feeder was recorded through the weighing module installed on the feeder. The specific flow rate of the feeder was then measured at different opening percentages, with six parallel measurements conducted for each condition to ensure accuracy. The average values were calculated to determine the feeding kinetics of the feeder, which describe the quantitative relationship between the feeder’s opening percentage and the resulting mass flow rate. Figure 4 illustrates the relationship between different opening percentages (x, %) and mass flow rates (y, g/min) of the volumetric feeder. This relationship can be expressed as a linear formula: y = 0.2843x − 0.5803. The linear correlation is strong, with a coefficient of determination of R^2^ = 0.9930, indicating high accuracy and reliability in the feeder’s performance under varying conditions.

**Figure 5:**
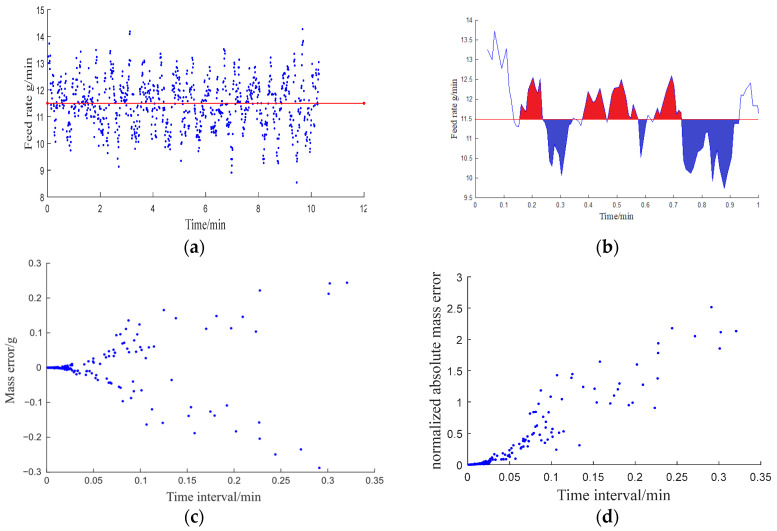
Feeding performance analysis of a volumetric feeder: (**a**) set feeding rate (red) and actual feeding rate (blue); (**b**) error analysis; (**c**) error funnel plot; (**d**) normalized error funnel plot.

The comparison between the set value of the feeding flow rate and the actual value (Figure 5a) shows that the actual feeding flow rate fluctuates around the set value. The deviation between the set and actual feeding flow rate curves indicates whether excessive (+) or insufficient (−) solid mass was injected compared to the set value during the actual operation of the feeder over a period of time (Figure 5b). Positive and negative feeding quality errors were segmented based on corresponding time intervals (Figure 5c). By normalizing the absolute quality error, the feeding performance under different feed rates can be compared. This normalization allows for a more intuitive evaluation of feeder accuracy and consistency. The data indicate that, in most cases, the maximum duration of deviation during the feeding process does not exceed 20 s. Additionally, the standardized feeding quality deviation generally remains below 2.5% (Figure 5d). These results demonstrate that the performance of the feeder satisfies the requirements for feeding speed and accuracy in continuous granulation.

#### 3.1.2. Calibration and Error Analysis of Liquid Feeding Rate

**Figure 6:**
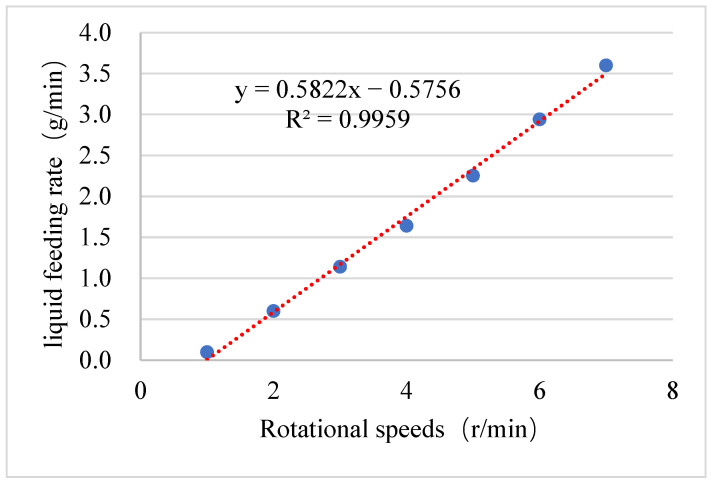
Relationship between different rotational speeds and the liquid feeding rate of the peristaltic pump.

Similarly, the accuracy and stability of the liquid feeding rate also have a significant impact on the granulation process. Therefore, it is essential to analyze the errors associated with the liquid feeding rate. Performance analysis was conducted on the peristaltic pump. The liquid flow rates of the peristaltic pump at different speeds were recorded using a balance to obtain the feed dynamics of the peristaltic pump. Figure 6 illustrates the relationship between different rotational speeds (x, r/min) and the liquid flow rate (y, g/min) of the peristaltic pump, represented by the following formula: y = 0.5822x − 0.5756. The linear correlation is strong, with R^2^ = 0.9959, indicating a reliable predictive capability of the pump’s performance at varying rotational speeds.

**Figure 7:**
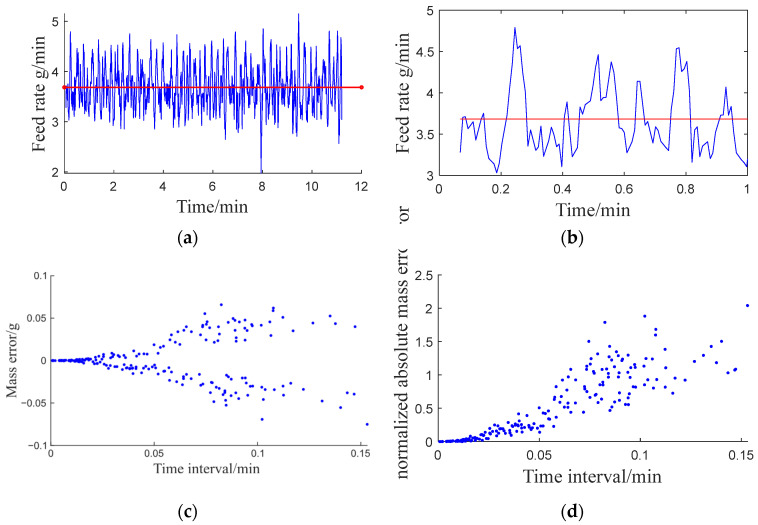
Analysis of the liquid feeding performance of the peristaltic pump: (**a**) the set liquid feeding rate (red) and actual feeding rate (blue); (**b**) error analysis; (**c**) error funnel plot; (**d**) normalized error funnel plot.

The variation of liquid mass over time was accurately obtained by setting the peristaltic pump speed to 7 r/min and recording the liquid mass data using a balance (Figure 7a). It was observed that the actual liquid inlet flow rate fluctuated around the set value. The deviation between the set liquid flow rate and the actual liquid flow rate curve reflects periods of excessive (+) or insufficient (−) liquid mass injection during the actual operation of the peristaltic pump (Figure 7b). These deviations are indicative of the pump’s dynamic performance under continuous operation. Segmented graphs of positive and negative errors in liquid mass addition were generated based on corresponding time intervals (Figure 7c). By normalizing the absolute mass error, the performance under different liquid inlet flow rates can be compared. This normalization enables a consistent evaluation of the pump’s performance across varying operating conditions. The graph shows that, in most cases, the maximum duration of deviation during the liquid inlet process is less than 9 s. Additionally, the standardized liquid mass deviation is mostly below 2.0% (Figure 7d), demonstrating high precision in liquid feeding. Hence, the performance of the peristaltic pump satisfies the requirements for continuous granulation, ensuring stable and accurate liquid delivery over time.

### 3.2. Calibration Between Tracer Concentration and Brightness Value

**Figure 8:**
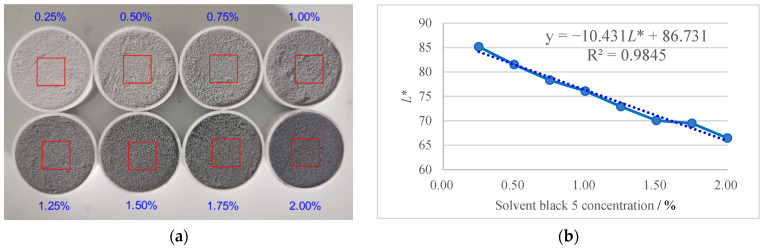
The relationship between tracer concentration and brightness value: (**a**) samples with different mass fractions of solvent black 5; (**b**) the relationship between tracers with different mass fractions and brightness values.

A calibration curve relating the concentration of solvent black 5 tracer to image lightness (L*) was established to facilitate quantitative analysis of the RTD. Samples with mass fractions of solvent black 5 ranging from 0.25% to 2.0% were prepared by adding accurately weighed amounts of solvent black 5 to a known mass of premix. The mixture was then thoroughly blended in a rotary mixer for 10 min to ensure uniform distribution of the tracer. The L* of each sample was measured using an image processing method. Figure 8a shows images of the prepared samples with different mass fractions of solvent black 5 ranging from 0.25% to 2.00%. Figure 8b presents the calibration curve between the tracer concentration and the corresponding lightness value (L*).

A strong linear correlation was observed between L* and the tracer concentration within the investigated range, with an R^2^ value of 0.9845. The relationship between the two is described by the following equation: C = −10.431L* + 86.731, where C is the mass fraction of solvent black 5 (wt%) and L* is the brightness value of the sample. This calibration curve was subsequently employed to determine tracer concentrations from L* values measured during the RTD experiments.

### 3.3. The Influence of Different Tracer Dosages on RTD

**Figure 9:**
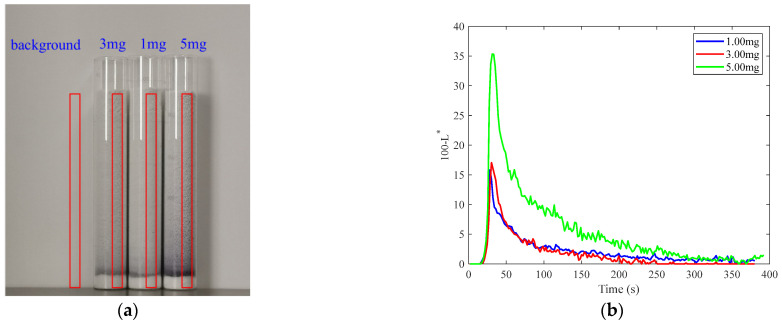
Distribution of residence time with different tracer dosages: (**a**) samples and detection areas corresponding to different tracer dosages; (**b**) brightness value curves corresponding to different tracer dosages.

The effect of the tracer dosage on the RTD was investigated to inform the selection of appropriate tracer dosages for subsequent experiments. In the TSWG experiment, the screw speed was set to 200 r/min, the powder feed rate was set to 13.2 g/min, the liquid feed rate was set to 1.8 g/min, and the barrel temperature was kept at 25 °C. After the granulation process stabilized for 10 min, the tracer was introduced at the feed inlet of the granulator, and granule samples were immediately collected from the discharge outlet. The start and end times of sample collection were recorded. Figure 9a shows the collected granule samples from TSWG and the corresponding detection areas for tracer dosages of 1 mg, 3 mg, and 5 mg. Each detection area has a width of 100 pixels and a height of from the bottom to the top of the granules.

The 100-L* curves for different tracer dosages are presented in Figure 9b. The 100-L* value increases with increasing tracer dosage. The difference in the 100-L* curves for 1 mg and 3 mg of tracer is small. At a tracer dosage of 5 mg, a notably higher 100-L* value is observed. This is further supported by the visually darker appearance of these regions compared to areas with less tracer dosage. The 100-L* curve corresponds to a 5 mg tracer and also exhibits a higher and broader peak, suggesting a longer residence time of the tracer.

**Figure 10:**
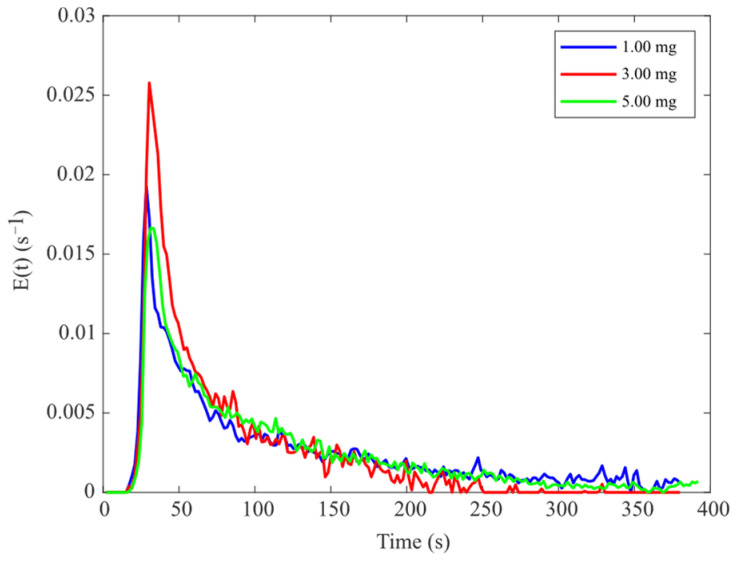
E(t) curves corresponding to different tracer dosages.

The E(t) curves of the samples corresponding to different tracer dosages are presented in Figure 10. The t_m_ and σ^2^ derived from these curves are summarized in Table 1. The data in Table 1 indicate that tracer dosage affects both t_m_ and σ^2^. As the tracer dosage increases, fluctuations in the E(t) curve decrease, resulting in smoother curves. This improved smoothness likely reduces errors associated with image analysis. Therefore, a tracer dosage of 5 mg was selected for subsequent experiments to maximize the accuracy of RTD measurements.

**Figure 11:**
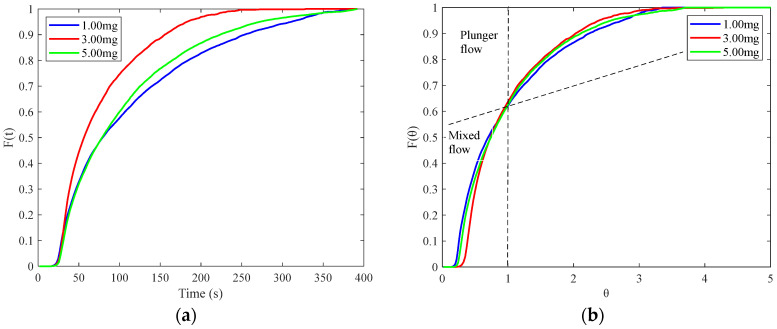
The RTD results corresponding to different tracer dosages: (**a**) F(t) curve; (**b**) F(θ) curve.

The F(t) curves for different tracer dosages are presented in Figure 11a. As the tracer dosage increases, the F(t) curve tends to become flatter. The dimensionless F(θ) curves for different tracer dosages are shown in Figure 11b. It can be seen from the figure that the F(θ) curves lie between the characteristics of plug flow and mixed flow. The small gaps between the curves suggest that variations in tracer dosage have a minimal impact on the mixing degree of materials, indicating that the tracer dosages meet the required specifications for RTD measurements.

### 3.4. The Influence of Different Screw Speeds on the RTD

**Figure 12:**
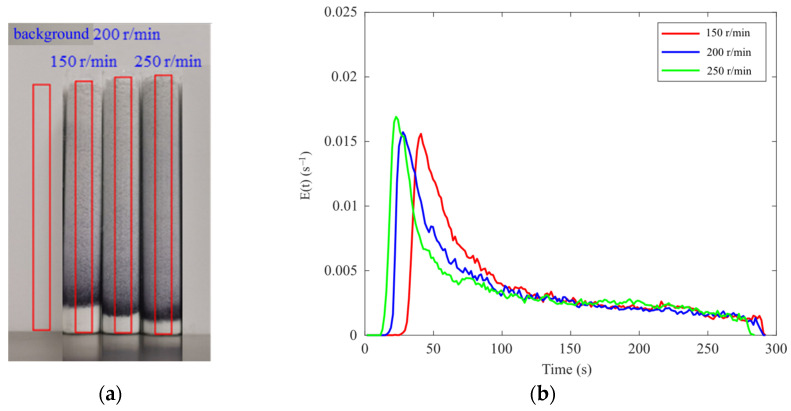
The RTD of samples with different screw speeds: (**a**) detection area; (**b**) E(t) curve.

In the TSWG experiment, the feeding opening percentage was kept at 60% with a powder feeding rate of 21.26 g/min. The speed of the peristaltic pump was set 6 r/min with a liquid feeding rate of 2.94 g/min, and the barrel temperature was maintained at 25 °C. The screw speeds were set to 150 r/min, 200 r/min, and 250 r/min. After a 10 min stabilization period of the granulation process, tracer was introduced through the feeder port of the granulator. Granule samples were immediately collected from the discharge port, and the collection start and end times were recorded. Figure 12a shows the collected granule samples and their corresponding detection areas for screw speeds of 150, 200, and 250 r/min. The detection area has a width of 100 pixels and a height of from the bottom to the top of the particles.

The E(t) curves for different screw speeds samples are shown in Figure 12b. Increasing the screw speed results in a higher peak value, a narrower width, and a leftward shift of the E(t) curve, indicating a decreased residence time and narrower distribution. The MRT t_m_ and its distribution domain (σ^2^) corresponding to different screw speeds are summarized in Table 2. As the screw speed increases, both the MRT and its variance decrease. A higher screw speed reduces the fill level in the barrel, lowering friction and material holdup, which decreases the MRT and narrows its variance [24]. This is consistent with the screw being the primary driver of material transport within the barrel, with a higher screw speed leading to faster material throughput and a shorter residence time [4,23].

**Figure 13:**
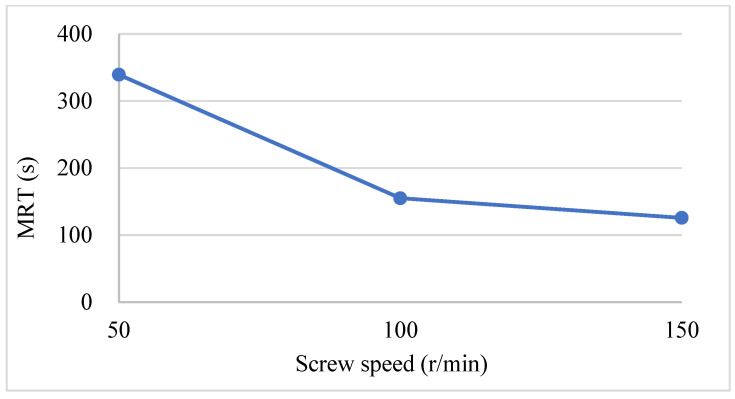
The relationship between MRT and screw speed.

Figure 13 shows the relationship between the screw speed and the MRT of RTD. The data demonstrate a negative correlation between screw speed and MRT under a constant powder and liquid feeding rate. As the screw speed increased from 150 r/min to 200 r/min, the MRT decreased from 111 s to 103 s. As the screw speed increased from 200 r/min to 250 r/min, the MRT decreased from 103 s to 102 s. Therefore, keeping other conditions constant, increasing the screw speed shortens the residence time. Conversely, decreasing the screw speed can prolong the residence time of materials, potentially enhancing the degree of material mixing and promoting granule formation and uniformity [25].

**Figure 14:**
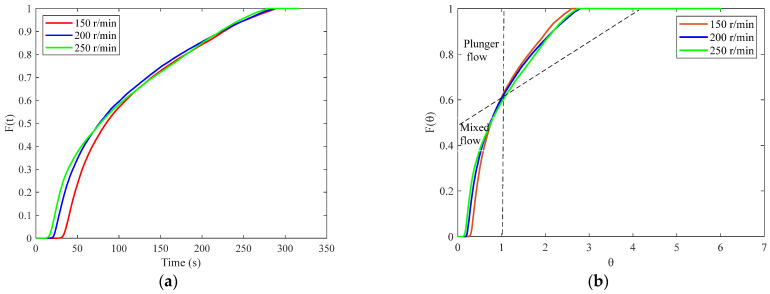
RTD results corresponding to different screw speeds: (**a**) F(t) curve; (**b**) F(θ) curve.

The F(t) and F(θ) curves for different screw speeds are shown in Figure 14a and Figure 14b, respectively. It can be seen that the curves exhibit characteristics between those of plunger flow and perfectly mixed flow. Increasing the screw speed shifts the curves closer to the behavior of a perfectly mixed flow, although the differences between the curves are relatively small, suggesting that the influence of screw speed on the degree of material mixing is limited within the investigated range.

### 3.5. The Influence of Different Throughputs on the RTD

**Figure 15:**
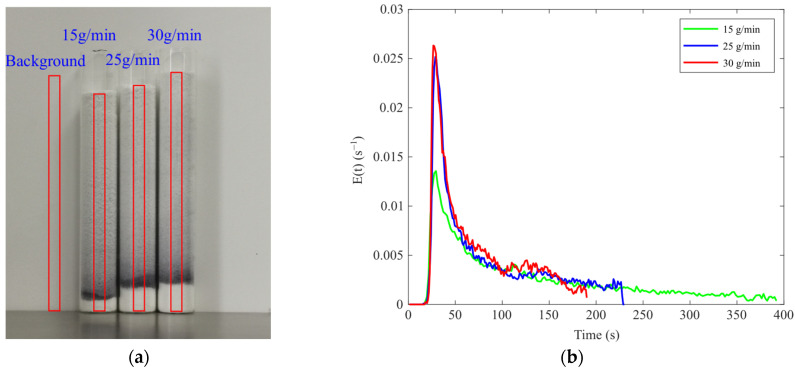
The RTD of samples with different throughputs: (**a**) detection area; (**b**) E(t) curve.

In this TSWG experiment, the screw speed was fixed at 200 r/min, the tracer dosage was 5 mg, and the barrel temperature was maintained at 25 °C. A constant liquid-to-solid (L/S) ratio of 0.14 was maintained by adjusting the opening percentage of the feeder and the rotational speed of the peristaltic pump. Experiments were conducted at feeder opening percentages of 40%, 60%, and 80%, corresponding to peristaltic pump speeds of 4.1 r/min, 6.0 r/min, and 7.5 r/min, respectively. After the granulation process was stabilized for 10 min for each throughput, tracer was introduced through the feeder inlet of the granulator, particle samples were immediately collected from the discharge outlet, and the collection start and end times of sample were recorded. Figure 15a shows the particle samples and detection areas obtained at different throughputs.

**Figure 16:**
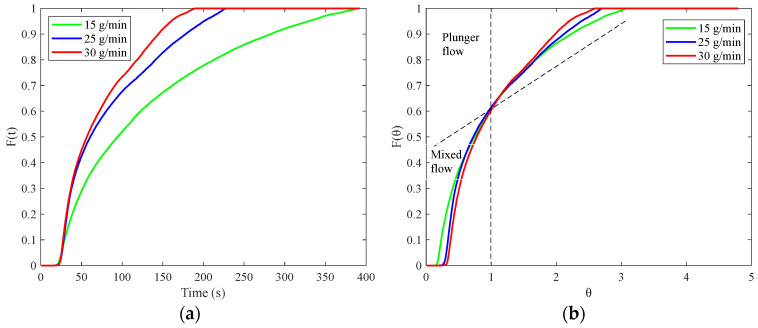
The RTD results corresponding to different throughputs: (**a**) F(t) curve; (**b**) F(θ) curve.

The E(t) curves for different throughputs are shown in Figure 16b. The peak value of the E(t) curve increases while the distribution narrows as the throughput increases. The t_m_ and σ^2^ values corresponding to different throughputs are presented in Table 3. Both the t_m_ and the σ^2^ decrease with the increasing throughput, consistent with the findings in the literature that the throughput affects the residence time of the material by influencing the filling level inside the barrel [25]. Increased throughput leads to a fill level of the material in the barrel, which, in turn, increases the conveying velocity of the material, thereby reducing both the MRT and its distribution, as reflected in the E(t) curves in Figure 16b.

## 4. Conclusions

The RTD parameters in the TSWG process are crucial for understanding the fundamental mechanisms of granulation and serve as a cornerstone for the development of control strategies in continuous granulation processes. This study addresses the limitations of traditional concentration detection methods in continuous granulation by introducing an innovative image-based RTD measurement method. The proposed method enables efficient measurement using a content measurement algorithm based on the Lab color space and facilitates comprehensive and precise evaluation of the impact of key process parameters, such as tracer dosage, screw speed, powder feeding rate, and liquid feeding rate, on MRT and variance within the TSWG process. Additionally, this method allows for image recognition during the granulation process, enabling real-time acquisition of RTD curves. This capability can be integrated with control systems to achieve real-time identification of abnormal materials, facilitating their diversion and ensuring product quality. The findings not only provide a deeper theoretical understanding of the mixing and transport phenomena in continuous granulation but also offer a robust and practical tool for process optimization and control strategies in the TSWG process.

## Figures and Tables

**Table 1:** The MRT and variance of different tracer dosages.

Tracer Dosages/mg	t_m_/s	σ^2^/s^2^	σ_θ_^2^
1.00	113	7939	0.62
3.00	77	2673	0.46
5.00	106	6396	0.57

**Table 2:** The MRT and variance parameters at different screw speeds.

Screw Speed (r/min)	t_m_/s	σ^2^/s^2^	σ_θ_^2^
150	111	4996	0.40
200	103	5451	0.52
250	102	5746	0.55

**Table 3:** The MRT for different throughput levels.

Opening Percentage of Volumetric Feeder (%)	Peristaltic Pump Speed (r/min)	L/S	Throughput (g/min)	t_m_/s	σ^2^/s^2^	σ_θ_^2^
40	4.1	0.14	10.79	127	9392	0.58
60	6	0.14	16.48	84	3327	0.47
80	7.5	0.14	22.16	73	2022	0.38

## Data Availability

The data presented in this study are available in this article.
